# Prospective study on the impact of BEAM versus FEAM conditioning on occurrence of neutropenic enterocolitis and on transplant outcome in lymphoma patients

**DOI:** 10.3389/fonc.2024.1369601

**Published:** 2024-05-13

**Authors:** Edoardo Benedetti, Ginevra Traverso, Giulia Pucci, Riccardo Morganti, Emilia Bramanti, Federica Cavallo, Enrico Capochiani, Maurizio De Maria, Vittorio Ricchiuto, Massimo Salvatore Stella, Sara Galimberti

**Affiliations:** ^1^ Operational Unit Hematology, Department of Clinical and Experimental Medicine, Azienda Ospedaliero Universitaria Pisana, Pisa, Italy; ^2^ Viale delle Milizie 9 00195 Roma, Italian School of Basic and Emergency Ultrasound Società Italiana di Ultrasonologia in Medicina e Biologia (SIUMB), Pisa, Italy; ^3^ Section of Statistics, Azienda Ospedaliero Universitaria Pisana, Pisa, Italy; ^4^ Institute of Chemistry of Organo Metallic Compounds (ICCOM), Consiglio Nazionale delle Ricerche (CNR), Pisa, Italy; ^5^ Division of Hematology U, University Hospital Azienda Ospedaliero Universitaria (AOU) “Città della Salute e della Scienza”, Turin, Italy; ^6^ Division of Hematology U, Department of Molecular Biotechnologies and Health Sciences, University of Turin, Torino, Italy; ^7^ Hematology Unit, Azienda Unità Sanitaria Locale (USL) Toscana Nord Ovest, Livorno, Italy; ^8^ Dipartimento di Tecnologie Sanitarie Ente di Supporto Tecnico Amministrativo Regionale (ESTAR), Azienda Ospedaliero-Universitaria Pisana, Pisa, Italy; ^9^ Hematology Unit, Department of Translational Research and New Technologies in Medicine and Surgery, University of Pisa, Pisa, Italy

**Keywords:** neutropenic enterocolitis, NEC, ultrasound sonography, beam, FEAM, intestinal toxicity, autologous stem cell transplantation

## Abstract

**Introduction:**

Carmustine (BCNU), etoposide, cytarabine, and melphalan (BEAM) are a widely used high-dose chemotherapy regimen for autologous stem cell transplantation transplant (ASCT) in lymphoid malignancies. During BCNU shortages, some centers switched to fotemustine-substituted BEAM (FEAM). Neutropenic enterocolitis (NEC) is a life-threatening complication occurring after intestinal mucosa damage related to intensive chemotherapy. NEC mortality may be up to 30%–50%. In our study, we compared NEC incidence, symptoms, mortality, and transplant outcome in terms of overall survival (OS) and progression-free survival (PFS) in the BEAM *vs*. FEAM groups. Furthermore, we compared the cost of hospitalization of patients who did *vs*. patients who did not experience a NEC episode (NECe).

**Methods:**

A total of 191 patients were enrolled in this study (N = 129 and N = 62 were conditioned with BEAM and FEAM, respectively). All patients received bed-side high-resolution ultrasound (US) for NEC diagnosis.

**Results and discussion:**

NEC incidence and NEC-related mortality were similar in the BEAM and FEAM groups (31% and 40.3%, p = 0.653, and 5% and 8%, p = 0.627, respectively). At a median follow-up of 116 months, no difference was noted between BEAM *vs*. FEAM groups in terms of OS and PFS (p = 0.181 and p = 0.978, respectively). BEAM appeared equivalent to FEAM in terms of NEC incidence and efficacy. The high incidence of NEC and the low mortality is related to a timely US diagnosis and prompt treatment. US knowledge in NEC diagnosis allows to have comparable days of hospitalization of patients NECpos *vs*. patients NECneg. The cost analysis of NECpos *vs*. NECneg has been also performed.

## 1 Introduction

High-dose chemotherapy and autologous stem cell transplantation (ASCT) are an established treatment in patients with Hodgkin (HL) and non-Hodgkin lymphoma (NHL) (1). BEAM is a highly effective conditioning regimen for autologous stem cell transplant (ASCT) in relapsed and refractory HL (2) and NHL (3). Most frequent extra hematological toxicities reported in carmustine- or bis-chloroethylnitrosourea (BCNU)-containing regimens are hepatotoxicity, nephrotoxicity, chemotherapy-induced diarrhea, nausea, vomiting, severe mucositis (3–6), and pulmonary complications (7). In 2010, an unexpected BCNU shortage (8) challenged physicians to replace BCNU. Fotemustine, a third-generation nitrosourea, was used as a substitute for BCNU. A FEAM-conditioning regimen showed promising results in 84 patients with NHL and HL in a retrospective study (9, 10). In two retrospective studies, FEAM showed a higher incidence of mucotoxicity compared with BEAM (11, 12). Conversely another study (9) described a favorable toxicity profile in patients conditioned with FEAM.

Neutropenic enterocolitis is a life-threatening complication of patients experiencing mucosal damage in chemotherapy-related neutropenia (13–21). Although the pathogenesis is multifactorial, a chemotherapy-related mucosal damage results in mucosal barrier leading to invasion of the bowel wall from GUT bacteria (13, 14, 16, 17). NEC early diagnosis and prompt treatment were shown to reduce mortality (19–22). NEC incidence has been previously described in 297 patients affected by NHL, HL, and multiple myeloma undergoing ASCT. Patients were transplanted with either BEAM or busulfan and cyclophosphamide (Bu/Cy2), and NEC occurred in 12% of patients (21).

In this study, we prospectively enrolled patients conditioned with either BEAM or FEAM to compare NEC incidence, symptoms, and NEC-related mortality. We also compared the transplant outcome, i.e., overall survival (OS) and progression-free survival (PFS), of patients transplanted with BEAM *vs*. FEAM. Furthermore, we compared the cost of hospitalization of patients who did *vs*. patients who did not experience a NEC episode (NECe).

## 2 Materials and methods

### 2.1 Patients and study design

From November 2002 to December 2022, all patients admitted in our BMT Unit, undergoing ASCT with diagnosis of either NHL or HL, and receiving either BEAM (21) or FEAM (9, 10) were prospectively enrolled in the study. The conditioning regimen changed twice over the time period of the study, because of shortage of carmustine (8). Thus, BEAM was used from 2002 throughout 2013, and then FEAM was used from 2014 throughout 2017, and then from 2018 until December 2022 BEAM was reintroduced. The study was conducted in the Division of Hematology and to the Bone Marrow Transplant Unit of the University of Pisa, Italy. All patients provided written informed consent, and the study was approved by the Ethical committee (study no. 3636 approved on 06/21/2012).

### 2.2 Conditioning regimens, supportive therapy, and prophylaxis

Patients were treated with either BEAM or with FEAM as previously described (9, 10, 21). Day 0 was considered the day of infusion of autologous stem cells. All patients received posttransplant granulocyte colony stimulating factor (filgrastim), which was started on the day after reinfusion (+1) and continued until neutrophil recovery. Antimicrobial prophylaxis consisted of oral fluconazole, ciprofloxacin, and acyclovir, started on day 0. Fluconazole and ciprofloxacin were stopped at hematologic recovery or administration of antimicrobial therapy to treat infection. Acyclovir was continued for 3 months after transplant. From January 2014, ciprofloxacin and fluconazole prophylaxis was discontinued (for center policy) but acyclovir prophylaxis was maintained. Cotrimoxazole was administered for *Pneumocystis jirovecii* pneumonia prophylaxis from hematologic recovery until 3 months after reinfusion. Patients were transplanted with unmanipulated grafts. Neutropenia and fever were described, and microbiological evaluation was performed as previously described (8) and in references therein (17, 19, 21). Routine rectal swabs were performed at patients’ admission before initiating conditioning regimen. Colonized patients were as defined previously (11).

### 2.3 Ultrasonographic examination

A bed-side US (B-US) was performed, as soon as patients were admitted on the ward, before receiving chemotherapy conditioning (19, 23, 24). B-US was repeated within 24 h from the onset of any symptom or combination of symptoms presented such as fever (F), and/or abdominal pain (AbdP) and/or diarrhea (D). Ultrasound was performed with an Esaote MyLab 25 ultrasonographer equipped with a 3.5–5.0-MHz convex probe and a 7.5-MHz linear transducer without any preparation (19). The entire gastrointestinal tract was examined as previously described (19, 20, 22, 23, 25, 26). The presence/absence of free abdominal fluid in all four quadrants and/or abdominal organ pathologies (17, 23, 24) was also assessed during each imaging study (25, 27, 28). Patients who were diagnosed with NEC (NECpos) were considered our study group. Asymptomatic patients received another bed-side US to assess the gastrointestinal tract after 5 days of neutropenia for trial purposes (17, 19, 20). Patients who did not experience a NEC episode (NECneg) during the entire observational period (from day 0 until discharge) were considered controls. Follow-up ultrasound was repeated if clinical conditions worsened or at onset of new symptoms. Ultrasound studies were performed either on weekdays or during the weekend as clinically indicated. In all patients, bed-side US was performed by a hematologist teacher at the Italian School for Basic and Emergency Ultrasound (SIUMB) at the University of Pisa, with expertise in GIUS ultrasound (18–20, 22, 27).

### 2.4 Definition of neutropenic enterocolitis

**Figure 1:**
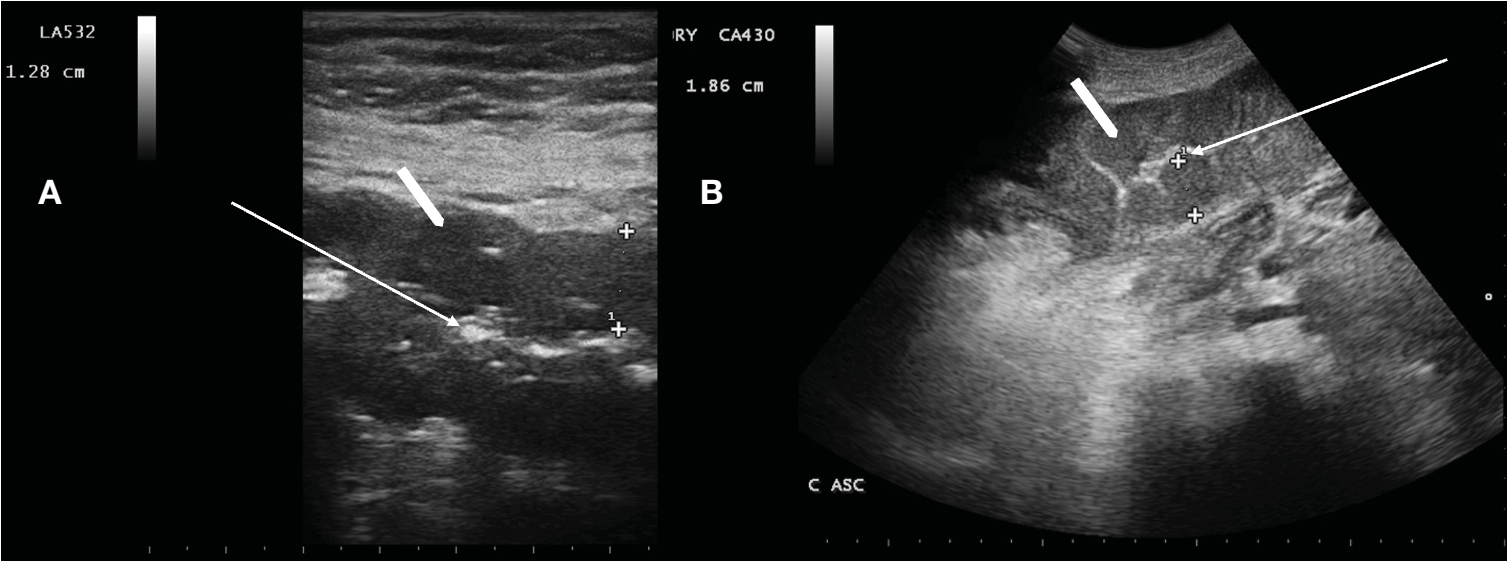
Neutropenic enterocolitis involving the transverse colon **(A)** and ascending colon **(B)** using a 3.5–5.0-MHz convex probe and a 7.5-MHz linear transducer, respectively. White arrow in A and B show compressed lumen. White arrowheads show that the bowel wall is abnormally thickened [**(A)** 12.8 mm, and **(B)** 18.6 mm]. Haustra are poorly recognizable.

NEC was defined as previously described. Using high-resolution US, the bowel wall thickness (BWT) greater than 4 mm for a length greater than 30 mm in the transverse scan was a key point for diagnosis (**Figure 1**) (16, 17, 19–21, 29, 30).

The axillary temperature ≥38.0°C was considered fever. Diarrhea was defined as more than three fluid stools/24 h, during neutropenia and neutropenia (16, 17, 31), which was defined as absolute neutrophil count (ANC) < 0.5 × 10^9^/L. Abdominal pain was evaluated using a Visual Analogous Scale Pain Score, ranging from 0 to 10 (17). NECe resolution was defined by the combination of the complete disappearance of symptoms and the normalization of all bowel segments involved at diagnosis with US (19, 20) (and references therein).

### 2.5 Treatment

As soon as NEC was diagnosed (clinical and ultrasonographic diagnosis), and blood cultures were obtained in febrile patients, a conservative approach with broad-spectrum antibiotics covering Gram- positive and -negative pathogens, anaerobes, and fungi was immediately started. Treatment included meropenem, liposomal ampho-B, and vancomycin. Caspofungin was used in patients with renal impairment and electrolyte imbalance. In sepsis/septic shock, IgM-enriched immunoglobulins were infused over 3 days (19, 20, 32, 33). Antibiotic treatment was modified according to sensitivity tests if infections were documented. The median duration of antimicrobial therapy for NEC was 5 days (range 3–9 days). All patients in the NEC cohort were treated conservatively, and none underwent surgery. Total parenteral nutrition, fluid resuscitation, transfusions of packed red blood cells, and platelet and fresh frozen plasma as needed (19–21) were used as part of the supportive treatment. NEC-related mortality was defined according to what was previously published (13, 14, 16, 17, 19, 21, 30, 34, 35).

### 2.6 Non-relapse mortality

We considered early non-relapse mortality (NRM) if the event of death occurred before discharge from the Hematology ward, prior to neutrophil engraftment. We considered late NRM if it occurred from discharge until day 100.

### 2.7 Statistical analysis

Categorical data were described by absolute and relative (%) frequency, continuous data by mean and standard deviation. Categorical and continuous patients’ characteristics were compared (BEAM *vs*. FEAM) using the chi-square test (or z-test for two proportions) and t-test for independent samples, respectively. Comparison between days of hospitalization and NEC (no, yes) was performed by t-test for independent samples. Survival curves were calculated with the Kaplan–Meier method and to evaluate the influence of the therapy (BEAM, FEAM) on the PFS and OS, univariate Cox regression was performed. Significance was set at 0.05, and all analyses were carried out with SPSS v.29 technology.

## 3 Results

### 3.1 Characteristics

From November 2002 to December 2022, 191 patients with NHL and HL who underwent ASCT were prospectively enrolled in this study. A total of 129 pts and 62 were conditioned with BEAM and FEAM, respectively. The patients’ characteristics are reported in **Table 1**. The two study populations (patients transplanted in the BEAM *vs*. FEAM groups) were homogeneous.

**Table 1:** Comparison between therapy (BEAM, FEAM) and patients characteristics.

Characteristic	BEAM	FEAM	p-value
**Age**	45(13)	46(12)	0.853
**BMI**	25.4 (4.6)	26.2 (4.6)	0.232
**Gender**			0.604
M	82	37	
	63.6%	59.7%	
F	47	25	
	36.4%	40.3%	
**Number of prior therapies**			0.108
1	23	5	
	17.8%	8.1%	
2	83	43	
	64.3%	69.4%	
3	21	10	
	16.3%	16.1%	
4	2	4	
	1.6%	6.5%	
**Status at transplant**			0.633
CR	102	48	
	79.1%	77.4%	
PR	23	11	
	17.8%	17.7%	
SD	1	2	
	0.8%	3.2%	
PD	3	1	
	2.3%	1.6%	
**DLBCL**			0.651
No	84	43	
	67.2%	70.5%	
Yes	41	18	
	32.8%	29.5%	
**FL**			0.705
**No**	106	53	
	84.8%	86.9%	
Yes	19	8	
	15.2%	13.1%	
**HL**			0.082
No	84	33	
	67.2%	54.1%	
Yes	41	28	
	32.8%	45.9%	
**MCL**			0.662
No	110	55	
	88.0%	90.2%	
Yes	15	6	
	12.0%	9.8%	

DLBCL, diffuse large B-cell lymphoma; HL, Hodgkin’s lymphoma; T-cell NHL, T-cell lymphoma; MCL, mantle cell lymphoma; PMBCL, primary mediastinal large B-cell lymphoma; CR, complete remission; PR, partial remission; SD, stable disease; PD, progressive disease; BMI, body mass index.

Statistics: mean and standard deviation or frequency and percentage.

### 3.2 NEC incidence

The overall incidence of NECe was 34% (65/191); 40/129 (31%) and 25/62 (40.3%) occurred in patients conditioned with BEAM and FEAM, respectively, without a statistically significant difference (p = 0.653). NEC was diagnosed at a median of 3.5 days of grade IV neutropenia (range 1–12 days). In January 2014, the internal antibiotic prophylaxis policy in patients undergoing ASCT changed, and ciprofloxacin prophylaxis was abandoned. The incidence of NEC before and after January 2014 was not statistically different (N = 33 NECe/112 ASCT before January 2014, *vs*. 32 NECe/79 ASCT thereafter, p = 0.113). The time to grade 4 neutropenia and its length in patients NECpos *vs*. patients NECneg were not significantly different within the BEAM and FEAM groups, as shown in **Table 2**. The median duration of symptoms was 5.5 days (range 3–9 days) and 4.5 days (range 3–8 days) in the BEAM and FEAM groups, respectively, without a statistically significant difference (p = 0.125).

**Table 2:** Number, type of cycles of CHT, number of NECe, and median length of days of neutropenia grade IV in AML patients during the study period (2007–2023).

	BEAM (NECpos)	BEAM (NECneg)	p value	FEAM (NECpos)	FEAM (NEC neg)	p value
Number of patients	40/129	89/129		25/62	37/62	
Time to neutropenia grade IV (median)	Day 2 (IQR 1–2)	Day 2 (IQR 0–2.5)	p = 0.925	Day 2 (IQR 1–3)	Day 2 (IQR 1–2)	p = 0.564
Length of neutropenia grade IV	Median 8 days (IQR7–8.5)	Median 7 days (IQR6–8)	p = 0.072	Median 7 days (IQR7–8)	Median 8 days (IQR7–9.5)	p = 0.555

### 3.3 NRM and NEC outcome

Overall, the non-relapse mortality (NRM) was 3.7% (7/191). Three patients died after discharge from the ward, at 2.5, 2, and 3 months, respectively (late NRM). The causes of NRM were pulmonary pneumonia acquired in the community (n = 1), multiorgan failure (MOF) (n = 1), and disseminated zoster virus (VZV) (n = 1). Four patients died during the neutropenic phase of the ASCT (early NRM), and in all of them, it was due to a NEC-related septic shock. Thus, overall mortality related to the occurrence of a NECe was 6% (4/65 NECe), NEC-related mortality was 5% (2/40 NECe), and 8% (2/25 NECe) in patients were conditioned with BEAM and FEAM, respectively. There was not a significant difference between NEC-related death occurring in the two groups (p = 0.627). The four NEC-related deaths occurred N = 1/4 (BEAM) in 2009, N = 1/4 in 2011 (BEAM), N = 1/4 in 2014 (FEAM), and 1/4 in 2015 (FEAM). From January 2015 (last patient deceased) up to December 2022, there were no other NEC-related deaths. There were 119 and 72 patients who were transplanted before and after January 2015, respectively; the NEC-related mortality (early NRM) was 11% (4/36) and 0% (0/29) before and after 2015, respectively, with a trend toward a statistical significance (p = 0.067).

### 3.4 Symptoms, intestinal involvement, and BWT

In **Table 3**, we report symptoms at NEC diagnosis. The most frequent symptoms were F+AbP+D, followed by F+D and Abd +D. Fever alone was never associated with NEC diagnosis in the entire cohort (N = 0/65). AP and D alone were found in 4.6% and 3%, respectively, at NEC diagnosis. The intestinal involved sites at NEC diagnosis were colon N = 33/65 (51%), ileum N = 17/65 (26%), and colon + ileum N = 15/65 (23%). In patients experiencing a NECe, the median BWT was 6.9 mm (range 4.5 mm–12.8 mm). In the four patients who died, NEC involved the ileum in N = 1/4 (BWT 11 mm) and the colon in N = 2/4 (BWT 10.2 mm and 12 mm, respectively), N = 1/4 last ileum loop plus caecum and ascending colon (maximum BWT 14 mm). BWT was higher in NECpos deceased (mean 11.8 mm stand deviation 1.6 mm) patients with respect to patients who survived a NECe (mean 7.1 mm standard deviation 2.1 mm), and the difference was statistically significant (p < 0.001). BWT was not found in the control group (NECneg). There were 64 out of 65 patients diagnosed with NEC who were treated with conservative medical treatment. One patient underwent surgery with partial ileum resection.

**Table 3:** Patient’s symptoms at NEC diagnosis.

Symptoms	Number	Percentage
Fever	0/65	0
Diarrhea	2/65	3%
Abdominal pain	3/65	4,6%
Fever + diarrhea	13/65	20%
Fever + abdominal pain	6/65	9.2%
Diarrhea+ abdominal pain	8/65	12.3%
Fever + diarrhea+ abdominal pain	33/65	50.7%

### 3.5 Infections

Microbiology-documented NEC infections (blood and/or stool) were found in 37% (24/65) NECe. Blood cultures were positive in 37% (24/65) and 15% (19/126) patients in the NECpos and NECneg groups, respectively (with a strong trend showing difference between NECpos *vs*. NECneg groups, P = 0.006). Gram-negative bacteria in blood cultures were the most represented, 46% (11/24) and 27% of which were MDR bacteria (3/11). Patients colonized were 17% (4/24). There was not a statistically significant impact of colonization (11) on the probability to experience a NECe (p = 0.722). In the 24 NECe with positive blood cultures, we detected the following pathogens: *Pseudomonas aeruginosa* (N = 3), two-thirds of which were quinolones, β-lactams, and aminoglycosides resistant; *Escherichia coli* (N = 4), among which one was β-lactamase (ESBL) resistant; *Klebsiella pneumoniae* (N = 3), two-thirds of which were non-carbapenemase-producing bacteria and one-third of which were carbapenemase-producing bacteria; *Campylobacter jejuni* (N = 1); *Candida albicans* (N = 2); *Candida krusei* (N = 1); *Enterococcus faecalis* (N = 4), *Staphylococcus haemolyticus* (N = 4), and *Staphylococcus aureus* (N = 2). All four patients who died of septic shock during a NECe had positive BC (N = 1 *KPC*; N = 2 multiresistant pseudomonas aeruginosa, N = 1 *E. coli*).

### 3.6 Survival

**Figure 2:**
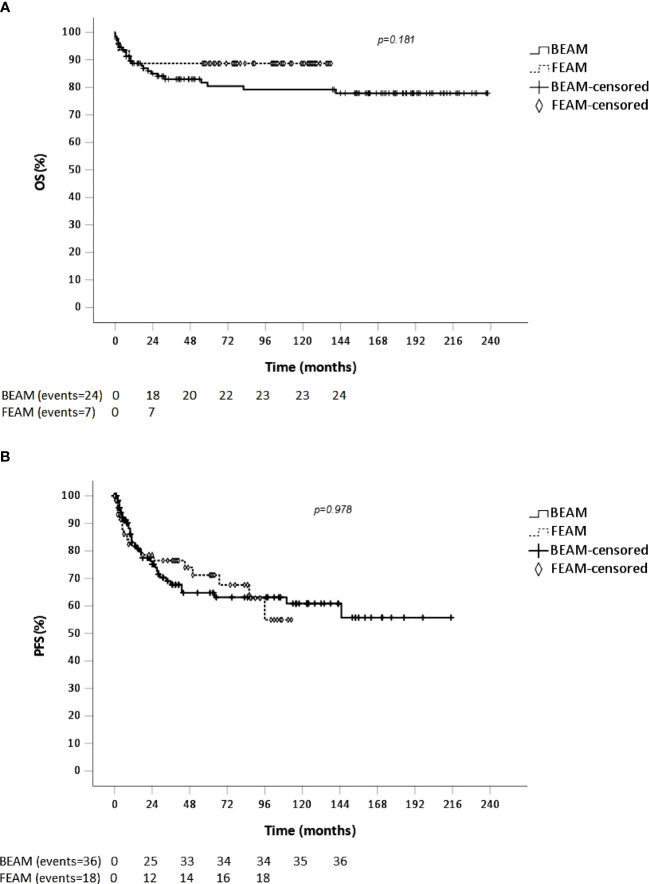
OS **(A)** and PFS **(B)** of patients conditioned with BEAM and FEAM (continuous line, cross symbol) and FEAM (dotted line, diamond signal).

In **Table 4**, we report the disease status at transplant. The BEAM and FEAM groups were homogeneously distributed. We analyzed if the following confounding variables had an impact on OS and PFS in the two groups (BEAM *vs*. FEAM), and we did not find a statistically significant impact: age p = 0.427; gender p = 0.604; BMI p = 0.116; histology diffuse large B-cell lymphoma (DLBCL) p = 0.651; Hodgkin’s lymphoma (HL) p = 0.082; mantle cell lymphoma (MCL) p = 0.662; follicular lymphoma (FL) p =0.705; number of lines of prior therapy pre-ASCT p = 0.108; and status of the disease at transplant p = 0.633. At a median follow-up of 116 months (range 3–238 months), the median OS and PFS were not reached in the whole cohort of patients, and there was not a statistically significant difference between BEAM and FEAM (p = 0.181 and p = 0.978, respectively, **Figures 2A, B**, respectively). The median follow-up of BEAM was longer (156 months, range 2-238 months) *vs*. FEAM (103 months, range 56–138 months).

**Table 4:** Patients’ symptoms at NEC diagnosis.

Status of the disease at Tx (number of patients)	Number of patients receiving BEAM = 129 (percentage)	Number of patients receiving FEAM = 129 (percentage)	*p* value
CR(150)	102(79)	48(77)	p = 0.754
PR(34)	23(18)	11(18)	p = 1.000
SD(3)	1(1)	2(3)	p = 0.310
PD(4)	3(2)	1(2)	p = 1.000

**Figure 3:**
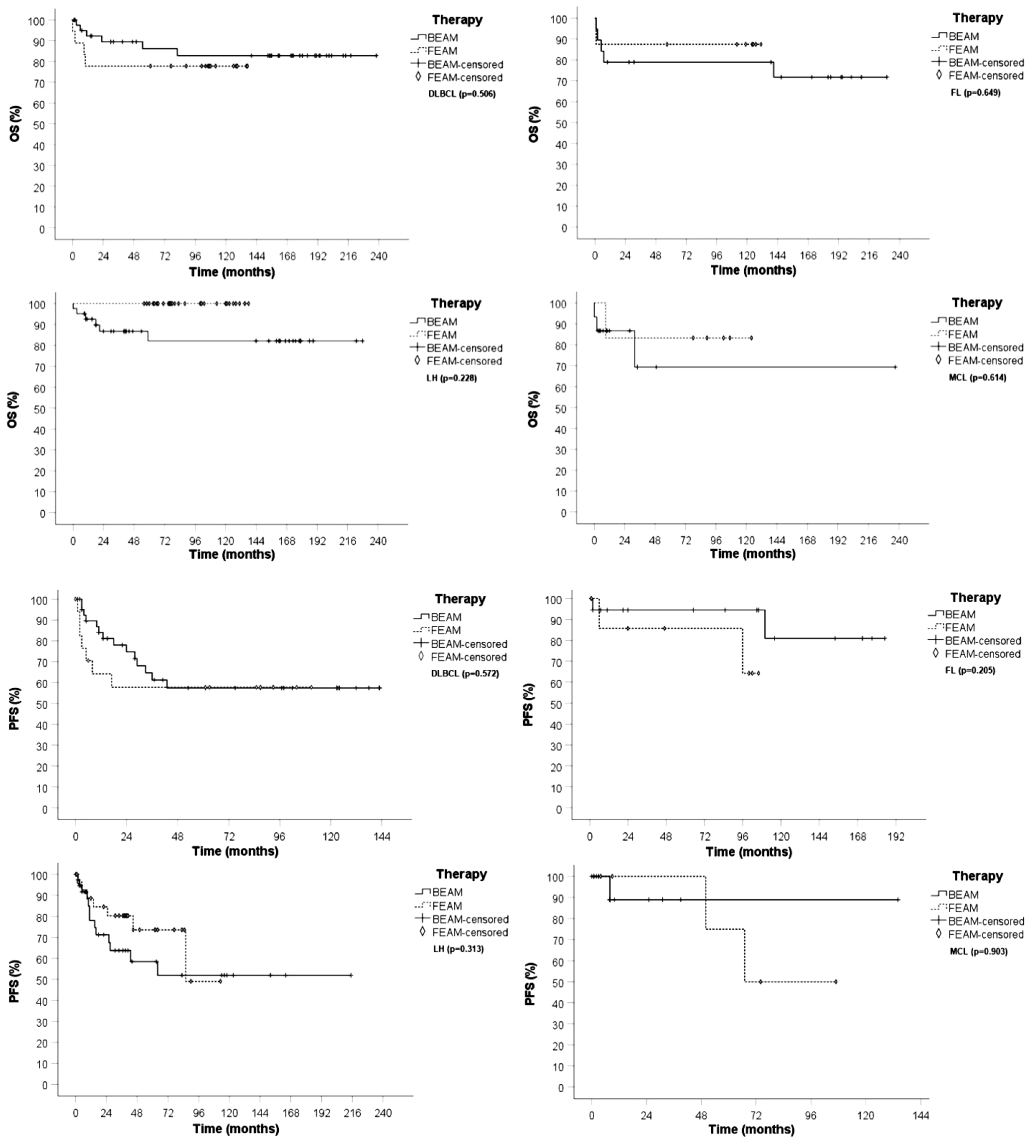
OS and PFS of patients conditioned with BEAM (continuous line, cross symbol) and FEAM (dotted line, diamond signal) in DLBCL,FL,HL and MCL.

We compared OS and PFS of BEAM *vs*. FEAM in the most represented histology (DLBCL, FL, HL, and MCL), and we found no statistically significant differences (**Figure 3**). Although median PFS in HL and in MCL in the FEAM group was reached at 90 months and 65 months, respectively, there was not a statistically significant difference compared with the BEAM group (**Figure 3**).

### 3.7 Costs

We analyzed the cost in euros of the management of patients experiencing a NECe compared with patients NECneg, calculating the days of hospitalization. The cost of a single room with HEPA filters and positive pressure is 1.470 euros per one day of hospitalization in the Tuscany Health Regional System. Overall patients who experienced NEC (NECpos group) have a median of hospitalization of 13 days post stem cell (SC) infusion (IQR = 11–17) *vs*. patients NECneg with a median of 13 days (IQR = 10–14). Despite that the median days of hospitalization was the same, the IQR distribution was statistically different (p = 0.024). In **Table 5**, we report the comparison of days of hospitalization of NECpos *vs*. NECneg patients, in different time periods because of change of conditioning regimens over time. We found a statistically significant difference between the two groups only in the period 2002–2013. The difference was not observed in the periods 2014–2017 and 2018–2022.

**Table 5:** Comparison of days of hospitalization of NEC pos vs. NECneg patients, in different time periods because of change of conditioning regimens over time stratified for period.

Period	Conditioning	NEC no	NEC yes	p-value
2002–2013	BEAM	12.1 (3.1)	14(5)	0.046
2014–2017	FEAM	15.2 (9.7)	16.9 (9.8)	0.548
2018–2022	BEAM	13.8 (9.3)	14.3(4)	0.811

Statistics: mean and standard deviation.

## 4 Discussion

High-dose chemotherapy (HDC) and autologous stem cell transplantation (ASCT) is a well-established treatment modality, which may offer a long-term disease control in patients with relapsed or refractory non-Hodgkin’s lymphoma (NHL) and Hodgkin’s lymphoma (HL) (36, 37). BEAM is a widely adopted conditioning regimen for ASCT, used since the 1990s (11). BEAM early adverse events related to BCNU include chemotherapy-induced nausea and vomiting, severe mucositis, hepatotoxicity, nephrotoxicity, and diarrhea (4–6, 11). Fotemustine is characterized by a reduced incidence of hepatic and renal complications and by the absence of pulmonary toxicity (38–40) with antitumor activity that is comparable with BCNU (9). NEC is a life-threatening complication occurring mainly in patients undergoing chemotherapy (13, 14, 16, 17, 19, 21, 35). The cytotoxic effect of chemotherapy can cause direct mucosal injury (13, 29, 34) with loss of gut barrier function, which may lead to a subsequent microbial invasion of the bowel wall by colonic organisms (41).

A previous multicenter retrospective study comparing BEAM *vs*. FEAM reported that FEAM resulted in a higher mucosal toxicity (11). To our knowledge, this is the first study with prospective enrolment of N = 191 patients, aimed to compare the incidence and outcome of intestinal mucosal damage leading to the life-threatening complication, namely, NEC, in BEAM- *vs*. FEAM-conditioning regimens in relapsed-refractory (RR) patients affected by NHL and HL. In this study, we found that NEC incidence was 31% and 40.3% in the BEAM and FEAM arms, respectively, without a statistically significant difference (p = 0.653) between the two arms. In the study from Gil et al. (11), the patients were conditioned with either BEAM or BuCy2, and the overall NEC incidence reported was 12%. The difference in NEC incidence between this study and our study might be attributable to the difference in conditioning regimens. Moreover, the study population Gil et al. enrolled NHL, HD, multiple myeloma, and acute myeloid leukemia patients (11). Previous studies (11, 12) comparing BEAM *vs*. FEAM differ from our study because they were retrospective multicenter studies, and they reported on oral mucosal toxicity and intestinal mucosal toxicity leading to diarrhea. In the study from Musso et al. (9), patients were conditioned with FEAM and mucositis-grade (G) G3/G4 was detected in 30% of cases, chemotherapy-induced nausea and vomiting. G2/G3 was documented in 47% of patients, without detection of G4 toxicity. Diarrhea G2 and G3 were found in 17% and 7%, respectively. Retrospective studies not aimed to detect NEC using a timely bed-side US (16, 17, 19, 21) might underestimate the rate of NEC. Moreover, the lack of robust literature of NEC-dedicated studies in the ASCT field and the lack of uniformly accepted definitions and diagnostic criteria for this life-threatening complication might also contribute to explain the difference with our study (30).

Our study was designed and focused to assess intestinal mucosal barrier injury by conditioning regimens (NECe), using high-resolution bed-side US. We found that modified antibiotic prophylaxis since 2014 had no statistically significant impact on the incidence of NEC. Furthermore, neither the time to grade 4 neutropenia and the length of grade 4 neutropenia nor the intestinal colonization had a statistically significant impact on the probability to develop a NECe. These findings lead to speculate that the mucosal barrier damage is mostly related to the direct damage due to chemotherapy, as shown in a previous study (20). In our study, we diagnosed a high incidence of chemotherapy-related intestinal mucosal injury in both conditioning regimens (BEAM and FEAM); nevertheless, overall NEC-related mortalities were 6%, 5%, and 8% in patients conditioned with BEAM and FEAM, respectively, without a statistical difference (p = 0.653). The low mortality in the two arms of the study is in accordance with what previously reported (NRM 2.4% with FEAM, and 0% to 11% with carmustine-based regimens (9)). Sharma et al. (36) reported day 100 TRM of 15.6% in the BEAM group and 12% in the FEAM group. Thus, although in our study we aimed at NEC-related incidence and mortality, our findings are in accordance with the mortality due to overall infections reported in BEAM and FEAM in the literature (9). Musso et al. described a favorable toxicity profile in 84 consecutive patients conditioned with FEAM (9). Conversely in the retrospective multicenter study from both Marchesi et al. (12) and Oliveri et al. (11), FEAM showed a higher incidence of G3/G4 oral mucositis, and clinically or microbiologically documented infection complications. In the study from Olivieri et al., FEAM determined a higher incidence of mucotoxicity related to different enteric mucosal damage (11). Is it noteworthy that the occurrence of diarrhea as a result from intestinal mucositis grade ≥3 was previously reported to be 42% in BEAM (42) and 15%–30% in FEAM (9, 10). This finding is comparable with the NEC incidence found in our study. The absence of reported NEC episodes in these studies with respect to our findings might be explained by the design of our prospective study, which was focused on an early detection of NEC using a prompt high-resolution bed-side US.

Microbiologically documented infections revealed that Gram-negative bacteria were the most common pathogens in accordance with that previously reported by Gil et al. (30). Overall, 27% were multidrug-resistant (MDR) bacteria. We did not find a correlation between the intestinal colonization and the probability to experience a NECe. We might explain this observation by the fact that in the NEC pathogenesis, the intestinal mucosal barrier has to be injured by chemotherapy to allow intestinal pathogens to reach the bowel wall layers causing the NEC syndrome (16, 19–21).

The analysis of symptoms at NEC diagnosis showed that fever alone was never present. Accordingly, this finding has been previously reported in two other studies (19, 20). The most involved intestinal site was colon, followed by ileum and colon + ileum.

It was previously reported that BWT has a prognostic significance (35). Due to relatively low incidence of early NRM in our study, we cannot draw any statistically significant conclusion; nevertheless, the patient who died had a BWT of >10 mm. Furthermore, our study shows that BWT is pathognomonic of NEC as it was never detected with US in the NECneg group according to previously reported studies (16, 19, 20).

We analyzed OS and PFS comparing the two conditioning regimens. We found no statistically significant difference in accordance with that previously reported (11, 12, 43), and furthermore, OS and PFS were comparable if patients were stratified for the most represented histology subtypes (DLBCL, MCL, FL) (**Figures 2**, **3**).

We analyzed the impact of a NECe on the length of hospitalization, and we found a statistically significant difference between NECpos and NECneg groups only in the time period from 2002 to 2013. Since 2014 in patients with timely diagnosis of NEC by US and immediate treatment, NECe did not result in a statistically significant increase in hospitalization days compared with the NECneg ASCT group, resulting in high cost savings. We can explain these data with the increased knowledge of timely ultrasound diagnosis focused on NEC followed by timely treatment. This explanation could be also supported by the absence of NEC-related deaths after 2015.

## 5 Conclusions

Our prospective NEC-oriented study shows that BEAM and FEAM condition regimens are comparable in terms of incidence of life-threatening intestinal mucosal injury (NECe), NEC-related mortality, OS, and PFS. The high incidence of NEC- and low NEC-related mortality may suggest that timely, high-resolution bedside ultrasound is a valid, repeatable, radiation-free tool that allows evaluation of critically ill neutropenic patients without leaving the isolation room.

## Data availability statement

The raw data supporting the conclusions of this article will be made available by the authors, without undue reservation.

## Ethics statement

The studies involving humans were approved by Comitato Etico Area Vasta Nord Ovest (CEAVNO) Toscana. The studies were conducted in accordance with the local legislation and institutional requirements. The participants provided their written informed consent to participate in this study.

## Author contributions

EdB: Conceptualization, Data curation, Formal analysis, Funding acquisition, Investigation, Methodology, Project administration, Resources, Software, Supervision, Validation, Visualization, Writing – original draft, Writing – review & editing. GT: Data curation, Investigation, Methodology, Writing – review & editing. GP: Data curation, Investigation, Methodology, Writing – review & editing. RM: Data curation, Formal analysis, Investigation, Methodology, Software, Validation, Writing – review & editing. EmB: Data curation, Formal analysis, Methodology, Software, Validation, Writing – review & editing. FC: Writing – review & editing. EC: Formal analysis, Investigation, Methodology, Writing – review & editing. MD: Writing – review & editing. VR: Software, Validation, Writing – review & editing. MS: Investigation, Methodology, Writing – review & editing. SG: Funding acquisition, Resources, Supervision, Writing – review & editing.

## References

[1] Chen Y-B Lane AA Logan B Zhu X Akpek G Aljurf M. Impact of conditioning regimen on outcomes for patients with lymphoma undergoing high-dose therapy with autologous hematopoietic cell transplantation. Biol Blood Marrow Transplant J Am Soc Blood Marrow Transplant. (2015) 21:1046–53. doi: 10.1016/j.bbmt.2015.02.005 PMC4426014 25687795

[2] Linch DC Winfield D Goldstone AH Moir D Hancock B McMillan A. Dose intensification with autologous bone-marrow transplantation in relapsed and resistant Hodgkin’s disease: results of a BNLI randomised trial. Lancet (London England). (1993) 341:1051–4. doi: 10.1016/0140-6736(93)92411-l 8096958

[3] Mills W Chopra R McMillan A Pearce R Linch DC Goldstone AH. BEAM chemotherapy and autologous bone marrow transplantation for patients with relapsed or refractory non-Hodgkin’s lymphoma. J Clin Oncol Off J Am Soc Clin Oncol. (1995) 13:588–95. doi: 10.1200/JCO.1995.13.3.588 7884420

[4] Jo J-C Kang BW Jang G Sym SJ Lee SS Koo JE. BEAC or BEAM high-dose chemotherapy followed by autologous stem cell transplantation in non-Hodgkin’s lymphoma patients: comparative analysis of efficacy and toxicity. Ann Hematol. (2008) 87:43–8. doi: 10.1007/s00277-007-0360-0 17710401

[5] Jantunen E Kuittinen T Nousiainen T. BEAC or BEAM for high-dose therapy in patients with non-hodgkin’s lymphoma? A single centre analysis on toxicity and efficacy. Leuk Lymphoma. (2003) 44:1151–8. doi: 10.1080/1042819031000083028 12916867

[6] Salar A Sierra J Gandarillas M Caballero MD Marín J Lahuerta JJ. Autologous stem cell transplantation for clinically aggressive non-Hodgkin’s lymphoma: the role of preparative regimens. Bone Marrow Transplant. (2001) 27:405–12. doi: 10.1038/sj.bmt.1702795 11313670

[7] Alessandrino EP Bernasconi P Colombo A Caldera D Martinelli G Vitulo P. Pulmonary toxicity following carmustine-based preparative regimens and autologous peripheral blood progenitor cell transplantation in hematological Malignancies. Bone Marrow Transplant. (2000) 25:309–13. doi: 10.1038/sj.bmt.1702154 10673703

[8] Tirelli U Berretta M Spina M Michieli M Lazzarini R. Oncologic drug shortages also in Italy. Eur Rev Med Pharmacol Sci. (2012) 16:138–9. 22338561

[9] Musso M Scalone R Marcacci G Lanza F Di Renzo N Cascavilla N. Fotemustine plus etoposide, cytarabine and melphalan (FEAM) as a new conditioning regimen for lymphoma patients undergoing auto-SCT: a multicenter feasibility study. Bone Marrow Transplant. (2010) 45:1147–53. doi: 10.1038/bmt.2009.318 19898504

[10] Musso M Messina G Di Renzo N Di Carlo P Vitolo U Scalone R. Improved outcome of patients with relapsed/refractory Hodgkin lymphoma with a new fotemustine-based high-dose chemotherapy regimen. Br J Haematol. (2016) 172:111–21. doi: 10.1111/bjh.13803 PMC5053328 26458240

[11] Olivieri J Mosna F Pelosini M Fama A Rattotti S Giannoccaro M. A comparison of the conditioning regimens BEAM and FEAM for autologous hematopoietic stem cell transplantation in lymphoma: an observational study on 1038 patients from fondazione italiana linfomi. Biol Blood Marrow Transplant. (2018) 24:1814–22. doi: 10.1016/j.bbmt.2018.05.018 29857196

[12] Marchesi F Capria S Giannarelli D Trisolini SM Ansuinelli M Caputo MD. BEAM vs FEAM high-dose chemotherapy: retrospective study in lymphoma patients undergoing autologous stem cell transplant. Bone Marrow Transplant. (2018) 53:1051–4. doi: 10.1038/s41409-018-0120-x 29440737

[13] Davila ML. Neutropenic enterocolitis: Current issues in diagnosis and management. Curr Infect Dis Rep. (2007) 9:116–20. doi: 10.1007/s11908-007-0006-3 17324348

[14] Davila ML. Neutropenic enterocolitis. Curr Treat Options Gastroenterol. (2006) 9:249–55. doi: 10.1007/s11938-006-0043-2 16901388

[15] Gorschlüter M Glasmacher A Hahn C Leutner C Marklein G Remig J. Severe abdominal infections in neutropenic patients. Cancer Invest. (2001) 19:669–77. doi: 10.1081/CNV-100106141 11577807

[16] Gorschluter M Mey U Strehl J Ziske C Schepke M Schmidt-Wolf IGH. Neutropenic enterocolitis in adults: systematic analysis of evidence quality. Eur J Haematol. (2005) 75:1–13. doi: 10.1111/j.1600-0609.2005.00442.x 15946304

[17] Gorschlüter M Marklein G Höfling K Clarenbach R Baumgartner S Hahn C. Abdominal infections in patients with acute leukaemia: a prospective study applying ultrasonography and microbiology. Br J Haematol. (2002) 117:351–8. doi: 10.1046/j.1365-2141.2002.03434.x 11972517

[18] Benedetti E Caracciolo F Lippolis P Bruno B Caramella D Cerri F. Neutropenic enterocolitis: prospective study on usefulness of ultrasound sonography for early diagnosis and to guide medical or surgical treatment. Bone Marrow Transplant. (2012) 47:S77.

[19] Benedetti E Bruno B Martini F Morganti R Bramanti E Caracciolo F. Early diagnosis of neutropenic enterocolitis by bedside ultrasound in hematological Malignancies: A prospective study. J Clin Med. (2021) 10:4277. doi: 10.3390/jcm10184277 PMC8468879 34575387

[20] Benedetti E Traverso G Pucci G Morganti R Bramanti E Lippolis P. Impact of different chemotherapy regimens on intestinal mucosal injury assessed with bedside ultrasound: a study in 213 AML patients. Front Oncol. (2023) 13:1272072. doi: 10.3389/fonc.2023.1272072 38023169 PMC10646482

[21] Pugliese N Salvatore P Iula DV Catania MR Chiurazzi F Della Pepa R. Ultrasonography-driven combination antibiotic therapy with tigecycline significantly increases survival among patients with neutropenic enterocolitis following cytarabine-containing chemotherapy for the remission induction of acute myeloid leukemia. Cancer Med. (2017) 6:1500–11. doi: 10.1002/cam4.1063 PMC5504336 28556623

[22] Benedetti E Lippolis PV Caracciolo F Galimberti S Papineschi F Pelosini M. Ultrasound findings guided a successful hemicolectomy in a leukemic patient with neutropenic enterocolitis. J Ultrasound. (2008) 11:97–101. doi: 10.1016/j.jus.2008.05.001 23396752 PMC3553330

[23] Atkinson NSS Bryant RV Dong Y Maaser C Kucharzik T Maconi G. WFUMB position paper. Learning gastrointestinal ultrasound: theory and practice. Ultrasound Med Biol. (2016) 42:2732–42. doi: 10.1016/j.ultrasmedbio.2016.08.026 27742140

[24] Hollerweger A Dirks K Szopinski K. Transabdominal ultrasound of the gastrointestinal tract . In: EFSUMB course book on ultrasound (2012). p. 233–71. Available at: http://www.kosmos-host.co.uk/efsumb-ecb/coursebook-transgit_ch08.pd http://www.kosmos-host.co.uk/efsumb-ecb/coursebook-transgit_ch08.pdf.

[25] Kuzmich S Howlett DC Andi A Shah D Kuzmich T. Transabdominal sonography in assessment of the bowel in adults. Am J Roentgenol. (2009) 192:197–212. doi: 10.2214/AJR.07.3555 19098201

[26] Serra C Menozzi G Labate AMM Giangregorio F Gionchetti P Beltrami M. Ultrasound assessment of vascularization of the thickened terminal ileum wall in Crohn’s disease patients using a low-mechanical index real-time scanning technique with a second generation ultrasound contrast agent. Eur J Radiol. (2007) 62:114–21. doi: 10.1016/j.ejrad.2006.11.027 17239555

[27] Benedetti E Bruno B McDonald GB Paolicchi A Caracciolo F Papineschi F. Prospective qualitative and quantitative non-invasive evaluation of intestinal acute GVHD by contrast-enhanced ultrasound sonography. Bone Marrow Transplant. (2013) 48:1421–8. doi: 10.1038/bmt.2013.65 23665821

[28] Hagiu C Badea R. Applicability of abdominal ultrasonography in inflammatory bowel diseases. J Gastrointestin Liver Dis. (2007) 16:205—209. 17592573

[29] Nesher L Rolston KVI. Neutropenic enterocolitis, a growing concern in the era of widespread use of aggressive chemotherapy. Clin Infect Dis. (2013) 56:711–7. doi: 10.1093/cid/cis998 23196957

[30] Gil L Poplawski D Mol A Nowicki A Schneider A Komarnicki M. Neutropenic enterocolitis after high-dose chemotherapy and autologous stem cell transplantation: incidence, risk factors, and outcome. Transpl Infect Dis. (2013) 15:1–7. doi: 10.1111/j.1399-3062.2012.00777.x 22862907

[31] Sachak T Arnold MA Naini BV Graham RP Shah SS Cruise M. Neutropenic enterocolitis: new insights into a deadly entity. Am J Surg Pathol. (2015) 39:1635–42. doi: 10.1097/pas.0000000000000517 26414225

[32] Berlot G Dimastromatteo G. Use of IgM and IgA-enriched immunoglobulins in the treatment of severe sepsis and septic shock. Clinical experience. Minerva Anestesiol. (2004) 70:735–9. 15516885

[33] Cui J Wei X Lv H Li Y Li P Chen Z. The clinical efficacy of intravenous IgM-enriched immunoglobulin (pentaglobin) in sepsis or septic shock: a meta-analysis with trial sequential analysis. Ann Intensive Care. (2019) 9:27. doi: 10.1186/s13613-019-0501-3 30725235 PMC6365591

[34] Bremer CT Monahan BP. Necrotizing enterocolitis in neutropenia and chemotherapy: a clinical update and old lessons relearned. Curr Gastroenterol Rep. (2006) 8:333–41. doi: 10.1007/s11894-006-0055-z 16836946

[35] Cartoni C Dragoni F Micozzi A Pescarmona E Mecarocci S Chirletti P. Neutropenic enterocolitis in patients with acute leukemia: prognostic significance of bowel wall thickening detected by ultrasonography. J Clin Oncol. (2001) 19:756–61. doi: 10.1200/JCO.2001.19.3.756 11157028

[36] Sharma A Kayal S Iqbal S Malik PS Raina V. Comparison of BEAM vs. LEAM regimen in autologous transplant for lymphoma at AIIMS. Springerplus. (2013) 2:489. doi: 10.1186/2193-1801-2-489 25674395 PMC4320155

[37] Appelbaum FR. Hematopoietic cell transplantation for non-hodgkin’s lymphoma: yesterday, today, and tomorrow. J Clin Oncol. (2008) 26:2927–9. doi: 10.1200/JCO.2007.15.7479 18565876

[38] Brandes AA Tosoni A Franceschi E Blatt V Santoro A Faedi M. Fotemustine as second-line treatment for recurrent or progressive glioblastoma after concomitant and/or adjuvant temozolomide: a phase II trial of Gruppo Italiano Cooperativo di Neuro-Oncologia (GICNO). Cancer Chemother Pharmacol. (2009) 64:769–75. doi: 10.1007/s00280-009-0926-8 PMC2717374 19169684

[39] Fabrini MG Silvano G Lolli I Perrone F Marsella A Scotti V. A multi-institutional phase II study on second-line Fotemustine chemotherapy in recurrent glioblastoma. J Neurooncol. (2009) 92:79–86. doi: 10.1007/s11060-008-9739-6 19018476

[40] Laquerriere A Raguenez-Viotte G Paraire M Bizzari J-P Paresy M Fillastre J-P. Nitrosoureas lomustine, carmustine and fotemustine induced hepatotoxic perturbations in rats: Biochemical, morphological and flow cytometry studies. Eur J Cancer Clin Oncol. (1991) 27:630–8. doi: 10.1016/0277-5379(91)90232-3 1828975

[41] Wach M Dmoszynska A Wasik-Szczepanek E Pozarowski A Drop A Szczepanek D. Neutropenic enterocolitis: a serious complication during the treatment of acute leukemias. Ann Hematol. (2004) 83:522–6. doi: 10.1007/s00277-003-0815-x 14658010

[42] Blijlevens N Schwenkglenks M Bacon P D’Addio A Einsele H Maertens J. Prospective oral mucositis audit: oral mucositis in patients receiving high-dose melphalan or BEAM conditioning chemotherapy—European blood and marrow transplantation mucositis advisory group. J Clin Oncol. (2008) 26:1519–25. doi: 10.1200/JCO.2007.13.6028 18268357

[43] Damaj G Cornillon J Bouabdallah K Gressin R Vigouroux S Gastinne T. Carmustine replacement in intensive chemotherapy preceding reinjection of autologous HSCs in Hodgkin and non-Hodgkin lymphoma: a review. Bone Marrow Transplant. (2017) 52:941–9. doi: 10.1038/bmt.2016.340 28112752

